# Exploring the neuroprotective role of artesunate in mouse models of anti-NMDAR encephalitis: insights from molecular mechanisms and transmission electron microscopy

**DOI:** 10.1186/s12964-024-01652-4

**Published:** 2024-05-14

**Authors:** Jingsi Liu, Yingyi Huang, Tinglin Qian, Jinyu Chen, Yuewen Ding, Zhaohui Lai, Xinghua Zhong, Mingjun Lai, Huili Zhang, Yuanyuan Wang, Honghao Wang, Yu Peng

**Affiliations:** 1 Department of Neurology, Nanfang Hospital, Southern Medical University, Guangzhou, 510515 China; 2 School of Traditional Chinese Medicine, Southern Medical University, Guangzhou, 510515 China; 3 Department of Neurology, Ganzhou People’s Hospital, Ganzhou, 341000 China; 4 Department of Neurology, Guangzhou First People’s Hospital, School of Medicine, South China University of Technology, Guangzhou, 510641 China

**Keywords:** anti-NMDAR encephalitis, Mitophagy, PINK1/PARKIN pathway, Artesunate

## Abstract

**Background:**

The pathway involving PTEN-induced putative kinase 1 (PINK1) and PARKIN plays a crucial role in mitophagy, a process activated by artesunate (ART). We propose that patients with anti-N-methyl-D-aspartate receptor (NMDAR) encephalitis exhibit insufficient mitophagy, and ART enhances mitophagy via the PINK1/PARKIN pathway, thereby providing neuroprotection.

**Methods:**

Adult female mice aged 8–10 weeks were selected to create a passive transfer model of anti-NMDAR encephalitis. We conducted behavioral tests on these mice within a set timeframe. Techniques such as immunohistochemistry, immunofluorescence, and western blotting were employed to assess markers including PINK1, PARKIN, LC3B, p62, caspase3, and cleaved caspase3. The TUNEL assay was utilized to detect neuronal apoptosis, while transmission electron microscopy (TEM) was used to examine mitochondrial autophagosomes. Primary hippocampal neurons were cultured, treated, and then analyzed through immunofluorescence for mtDNA, mtROS, TMRM.

**Results:**

In comparison to the control group, mitophagy levels in the experimental group were not significantly altered, yet there was a notable increase in apoptotic neurons. Furthermore, markers indicative of mitochondrial leakage and damage were found to be elevated in the experimental group compared to the control group, but these markers showed improvement following ART treatment. ART was effective in activating the PINK1/PARKIN pathway, enhancing mitophagy, and diminishing neuronal apoptosis. Behavioral assessments revealed that ART ameliorated symptoms in mice with anti-NMDAR encephalitis in the passive transfer model (PTM). The knockdown of PINK1 led to a reduction in mitophagy levels, and subsequent ART intervention did not alleviate symptoms in the anti-NMDAR encephalitis PTM mice, indicating that ART’s therapeutic efficacy is mediated through the activation of the PINK1/PARKIN pathway.

**Conclusions:**

At the onset of anti-NMDAR encephalitis, mitochondrial damage is observed; however, this damage is mitigated by the activation of mitophagy via the PINK1/PARKIN pathway. This regulatory feedback mechanism facilitates the removal of damaged mitochondria, prevents neuronal apoptosis, and consequently safeguards neural tissue. ART activates the PINK1/PARKIN pathway to enhance mitophagy, thereby exerting neuroprotective effects and may achieve therapeutic goals in treating anti-NMDAR encephalitis.

**Supplementary Information:**

The online version contains supplementary material available at 10.1186/s12964-024-01652-4.

## Introduction

Anti -NMDAR encephalitis is the most common autoimmune encephalitis [1], which was first discovered and reported by Josep Dalmau and his team in 2007. It presents with severe acute symptoms and results in a characteristic, serious neuropsychiatric syndromes [2, 3]. Current first-line therapy regimens typically is immunotherapy, including intravenous immunoglobulin(IVIG), steroids or plasmapheresis to remove pathogenic disease-causing autoantibodies, the second-line treatment is monoclonal antibody rituximab to unselectively deplete CD20^+^ B cells [3]. However, such immunotherapy may thus cause associated with side effects, such as septic shock, life-threatening sepsis as well as pulmonary embolism resulting in treatment interruption [4]. Therefore, it is necessary to develop new drugs to address this issue. Anti-NMDAR encephalitis is generally regarded as a form of neuroinflammation, though the current understanding of its pathogenesis remains unclear [5]. Despite comprising only 2% of human body weight, the adult brain is responsible for 20% of the body's resting metabolic rate, with a high demand for metabolic energy [6, 7]. These high energy demands also render the brain vulnerable to damage during periods of anoxia or ischemia [8, 9]. Mitochondria are the center of aerobic metabolism, with neurons relying predominantly on mitochondrial oxidative phosphorylation to generate ATP. Consequently, the importance of mitochondria and related pathways is undeniable. The degradation of damaged organelles and cytoplasmic components leads to mitophagy. Consequently, mitophagy plays a vital role in the central nervous system [8, 9]. Emerging evidence has revealed that mitophagy is closely associated with neuroinflammation in research into various neurodegenerative diseases, such as Parkinson’s Disease (PD), Alzheimer's Disease (AD), Huntington's Disease (HD), and Amyotrophic Lateral Sclerosis (ALS) [9–12]. Increasingly, new evidence suggests a causal relationship between impaired mitophagy and neurodegenerative diseases such as Alzheimer's Disease (AD) and Parkinson's Disease (PD) and other neurodegenerative pathologies. Therefore, it can be inferred that as a form of neuroinflammation, anti-NMDAR encephalitis is closely associated with mitophagy.

In 2008, research by Youle and his colleagues demonstrated that recruiting PARKIN to depolarize mitochondria promotes their autophagic degradation [13], which was regarded as a milestone in the field of mitophagy. Since then, research into mitophagy has evolved considerably, uncovering numerous pathways involved in mitophagy. These pathways are divided into ubiquitin-dependent and ubiquitin-independent mechanisms. The former includes PTEN-induced putative kinase 1 (PINK1) -PARKIN mediated mitophagy and PARKIN-independent pathways reliant on alternative ubiquitination processes. Mitophagy-related factors within the ubiquitin-independent pathway include NIX, BNIP3, BNIP3L, FUNDC1, FKBP8, BCL2L13, AMBRA1, PHB2, Cardiolipin, Ceramide, MUL1, SIAH1, ARIH1 [14]. Additionally, the cGAS-STING pathway is also associated with mitophagy [15–17]. Among the most characterized mechanisms is the PINK1-PARKIN mediated mitophagy, which has been extensively studied in the realm of neurodegenerative diseases. The serine/threonine kinase known as PINK1 is encoded by the *Park6* gene, and PARKIN, an E3 ubiquitin ligase, is encoded by the *Park2* gene [18]. It is widely acknowledged that mutations in *Pink1* and *Parkin* represent some of the earliest genetic events linked with autosomal recessive early-onset Parkinson's disease [18, 19]. This suggests that the PINK1/PARKIN pathway plays a critical role in the mitophagy of neuronal cells.

ART is a water-soluble derivative of artemisinin and intravenous ART is first-line therapy for severe malaria and cerebral malaria [20–22]. It is a prodrug that is rapidly metabolized under biological conditions into its active form, dihydroartemisinin (DHA) [23–27]. Previous research revealed that the mechanism that ART induces mitophagy through the PINK1-dependent pathway is to facilitate the stabilization of the full-length version of PINK1 on the mitochondria, while concurrently activating the pathway dependent on PINK1 [28]. This activation subsequently initiates the recruitment of Parkin, sequestosome 1 (SQSTM1), ubiquitin, and the microtubule-associated proteins 1A/1B light chain 3 (LC3) within the mitochondria, ultimately resulting in the induction of mitophagy [28]. Considering the notable efficacy and safety profile of ART derivatives in the treatment of malaria, coupled with their ability to effectively traverse the blood–brain barrier, we have identified ART derivatives as the most promising candidates for the treatment of anti-NMDAR encephalitis. This study is designed to evaluate the feasibility of using ART derivatives in the treatment of anti-NMDAR encephalitis and to investigate the mechanism of action.

## Materials and methods

### Antibodies and special reagents

In this study, we utilized a selection of antibodies, including anti-PTEN-induced putative kinase 1 (PINK1) (23274–1-AP), anti-caspase3 (19677–1-AP), anti-p62 (18420–1-AP), and anti-GAPDH (10494–1-AP) obtained from Proteintech (Wuhan, China); anti-cleaved caspase-3 (9661 T) sourced from Cell Signaling Technology Inc. (Massachusetts, USA); anti-LC3B (NB100-2220) from Novus; anti-Parkin (sc-32282) and anti-dsDNA (sc-58749) from Santa Cruz Biotechnology, Inc. (Texas, USA); anti-GluN1 procured from Synaptic Systems GmbH (Göttingen, Germany); anti-MAP2 (M4403) from Sigma-Aldrich (St. Louis, MO, USA); and anti-β-actin from ABclonal (Wuhan, China). Additional materials included MitoSOX Red (#M36008), MitoTracker (M22426), tetramethylrhodamine (TMRM, I34361), and the Melon Gel IgG Spin Purification Kit (45,206) from Thermo Fisher Scientific, Inc. (Waltham, MA, USA); the B-27 Plus Neuronal Culture System (A3653401) from Gibco (Carlsbad, CA, USA) from Beyotime (Shanghai, China). Secondary antibodies were acquired from Abcam (Cambridge, UK). ART was sourced from Guilin Pharmaceutical Co., Ltd. (Guilin, China). Additionally, an adeno-associated virus (AAV) encoding *sh-Pink1* was designed and produced by OBiO Technology Corp., Ltd. (Shanghai, China). This selection of reagents and materials was critical for conducting the experiments detailed in our study, ensuring high specificity and reliability in our findings.

### Cell culture

As outlined in prior research, hippocampal neurons were isolated from rat pups on embryonic days 16 to 18 (E16-18) [29, 30]. These neurons were then cultured on glass coverslips after being dissociated and maintained in a neuronal culture medium. This medium comprised neurobasal medium (Thermo Fisher, 21,103,049), 2 mM GlutaMax (Thermo Fisher, 35,050,061), B27 supplement (Thermo Fisher, 17504044), and 100 U/mL penicillin/streptomycin (Thermo Fisher, 15140122). On the 14th day in vitro, neurons received treatment with cerebrospinal fluid (CSF) from either patients anti-NMDAR encephalitis or control subjects at a concentration of 4 µg/ml, along with concurrent administration of ART or a vehicle control at concentrations of 1 µg/ml, 2 µg/ml, and 4 µg/ml for 24 h. Additionally, the human neuroblastoma cell line SH-SY5Y (ATCC, MD, U11SA) was cultured in DMEM/F12 medium enriched with 15% reduced-serum Opti-MEM (Gibco, CA, USA), 10% fetal bovine serum (Gibco, CA, USA), and 1% penicillin/streptomycin (100 U/mL), and incubated at 37 °C in an atmosphere containing 5% CO_2_. For ART treatment protocols, SH-SY5Y cells, once reaching 70–80% confluence, were prepared in reduced-serum Opti-MEM (Gibco, CA, USA) for 24 h. Following this preparation, cells were exposed to reduced-serum Opti-MEM (Gibco, CA, USA) supplemented with 8 μg/ml anti-NMDAR antibodies derived from patients and 4 μg/ml ART for durations of 0, 12, 24, or 48 h.

### Animals

Female C57BL/6 J mice, aged eight to ten weeks, were acquired from SPF Beijing Biotechnology Corporation (Beijing, China). These mice were housed in the specific pathogen-free conditions of the Laboratory Animal Center at Nanfang Hospital, Southern Medical University (Guangzhou, Guangdong, China), under a 12-h light–dark cycle, with unrestricted access to water and food. All procedures involving animals were performed in strict accordance with the guidelines and approval of the Animal Ethics Committee ofNanfang Hospital, Southern Medical University.

### CSF samples from patients and controls

CSF samples were collected from a randomly selected group of patients with anti-NMDAR encephalitis, comprising 10 females and 8 males, matched by age. The presence of NMDAR antibodies in these samples was confirmed based on the results of previous studies [31]. Control CSF samples were obtained from patients with non-inflammatory neurological disorders, such as cervical spondylosis. Both patient and control CSF samples were processed using the Melon Gel IgG Spin Purification Kit (Thermo Fisher Scientific, 45206) and subsequently stored at -80 °C until further analysis. Participants provided written informed consent for the use of their CSF samples in this study and for the publication of related case details. This research received ethical approval from the Ethics Committee of Guangzhou First People's Hospital (approval No. K-2023–087-01).

### Experimental grouping and induction of the NMDAR mouse model

To reduce variability, female mice were selected for this investigation. Random assignment of the mice was achieved using the random number generator function in SPSS software (IBM Corporation, Armonk, NY, USA). Female C57BL/6 J mice, aged 8–10 weeks and weighing 18–22 g, were organized into eight distinct groups: NC + PBS (*n* = 17), NC + ART (*n* = 19), PTM + PBS (*n* = 16), PTM + ART (*n* = 15), sham + PBS + sh-vector (*n* = 11), PTM + PBS + sh-vector (*n* = 11), PTM + ART + sh-vector (*n* = 11), and PTM + ART + sh-PINK1 (*n* = 11). The care and examination of the mice were in strict adherence to the with the American Research Institute (ARRIVE) guidelines. All data collection was performed by an individual blind to the IgG administration status of the animals. Under anesthesia with isoflurane, mice were secured in a stereotaxic apparatus, and a 2-cm incision was made on the cranial surface. A subcutaneous pocket was then formed at the back of the mouse via blunt dissection, into which a subcutaneous osmotic pump (Alzet, model 2002) was inserted. This setup facilitated a continuous CSF infusion (volume, 200 μl; flow rate, 0.5 μl/h for 14 days; Alzet, Cupertino, CA), as has been described previously [32]. The coordinates for lateral ventricle injection were AP: -0.22 mm, ML: ± 0.95 mm, DV: -2.3 mm. Fourteen days post intraperitoneal injection of either ART or PBS, the mice were euthanized, and their brain tissues were collected for subsequent analysis. The animal study was bifurcated into two phases, employing four groups in each phase. The initial phase involved: mice receiving CSF from patients, with or without anti-NMDAR antibodies (diluted to 2 mg/dl in 200 μl of PBS in the infusion pump), treated with either ART (100 mg/kg, twice daily for 14 days) or an equivalent volume of phosphate-buffered saline (PBS). A week prior to modeling, bilateral injections of AAV vectors carrying sh-PINK1 and empty vectors were administered into the hippocampal CA1 region of intervention and control mice, respectively. The subsequent phase utilized four additional groups: The sham + PBS + sh-vector group was administered CSF from patients lacking anti-NMDAR antibodies, alongside an identical volume of PBS. The PTM + PBS + sh-vector group received CSF from patients treated with anti-NMDAR antibodies and PBS. The PTM + ART + sh-vector group was treated with CSF from patients with anti-NMDAR antibodies and ART (100 mg/kg, twice daily for 14 days). The PTM + ART + sh-PINK1 group received CSF from patients with anti-NMDAR antibodies and ART. Animal well-being was closely monitored by observing weight, physical condition, wound healing, and both spontaneous and induced behaviors throughout the experiment. All animal procedures were executed in compliance with the ARRIVE guidelines and the stipulations of the Animals (Scientific Procedures) Act 1986 in the UK. The study received endorsement from the Laboratory Animal Ethics Committee of Nanfang Hospital, Southern Medical University (Guangzhou, Guangdong, China). The experimentation was conducted blindly, with researchers unaware of the specific treatment conditions or group allocations.

### Behavioral tests

Throughout the entirety of the experimental process, researchers remained blind to the group assignments and treatments administered to the mice. On the 14th day following ART administration, a comprehensive series of behavioral assessments were conducted, adhering to a consistent schedule from 9:00 AM to 6:00 PM. Behavioral observations of the mice were meticulously documented utilizing a video tracking system (TopScan 3.0, Clever Sys Inc. USA).

#### Open-field test

Mice were placed in a square box (40 × 40 × 40 cm^3^), Jiliang Software Technology Co., Ltd., Shanghai, China), where they moved freely without any disturbances. The entire process was recorded for 10 min with camera. In addition, the TopScan video tracking software version 3.0 (Clever Sys, Virginia, USA) was used to record the total time spent in the center of the field (15 × 15 cm) and peripheral fields within 10 min. Following each experiment, the square box was meticulously cleaned with 75% alcohol to eliminate any potential contamination from urine or feces previously left by the mice.

#### Three-chamber social test

The rectangular three-chamber apparatus consisted of three chambers and the size of each chamber was 20 cm (length) × 40 cm (width) × 20 cm (height). Mice were placed in it and moved freely for 10 min to be acclimatized within the apparatus. Experimental mice were initially secured in a central chamber, with two metal cages positioned at either end of the chamber. A same-sex strange mouse was placed in one of these metal cages, and the experimental mice were then allowed to move freely; observations were immediately recorded for a duration of 10 min. Following this, the experimental mice were once again restrained in the central chamber, after which another same-sex strange mouse was introduced into the metal cage on the opposite end. The experimental mice were allowed free movement again, with observations promptly recorded for another 10 min. Following each experiment, the square box was meticulously cleaned with 75% alcohol to eliminate any potential contamination from urine or feces previously left by the mice.The time spent sniffing the two strange mice was analyzed using Topscan 3.0.

#### Novel object recognition test

We prepared a square box (40 × 40 × 40 cm^3^. On the first day, two identical objects were placed and secured on opposite sides of the chamber, each positioned 5 cm away from the chamber walls. Mice were then allowed to freely explore the chamber for a period of 10 min, with all activities being recorded. On the following day, during the same time period, one of the objects previously placed in the chamber was replaced with a different object. The mice were subsequently permitted to freely explore the chamber again for an additional 10 min and were recorded. Following each experiment, the square box was meticulously cleaned with 75% alcohol to eliminate any potential contamination from urine or feces previously left by the mice.

#### Elevated plus-maze test

The elevated plus maze consisted of two open arms (28 × 5 × 20 cm), two closed arms (28 × 5 cm), and a central area. Mice were placed in one of the open arms, facing the central zone, and their behavior was continuously monitored and recorded over a period of 5 min. The time spent in the open and closed arms and the number of entries per arm were recorded. Following each experiment, the square box was meticulously cleaned with 75% alcohol to eliminate any potential contamination from urine or feces previously left by the mice.

### Histopathology

For standard analysis, brain tissues from mice were harvested on the 22nd day following immunization, fixed in 4% paraformaldehyde, and subsequently embedded in paraffin. Hippocampal sections, each 5 μm in thickness, were prepared and subjected to hematoxylin and eosin (H&E) staining. Immunohistochemistry was conducted in alignment with established protocols [33]. Initially, the paraffin-embedded sections were deparaffinized and rehydrated. To inhibit endogenous peroxidase activity, sections were treated with 3% H_2_O_2_ before undergoing antigen retrieval in sodium citrate buffer, heated until boiling. Once cooled to room temperature, sections were rinsed in phosphate-buffered saline with Tween 20 (PBST) and blocked with 10% fetal bovine serum (10270–106; Gibco, USA) at 37 °C for an hour to prevent nonspecific antibody binding. The sections were then incubated overnight at 4 °C with primary antibodies directed against PINK1 (23274–1-AP; Proteintech, China), Parkin (sc-32282; Santa Cruz Biotechnology), LC3B (NB100-2220; Novus, USA), and p62 (18420–1-AP, China). The following day, after washing off excess primary antibodies with PBST, sections were incubated with a goat anti-rabbit secondary antibody (ab7090; Abcam, USA) at 37 °C for an hour. Any remaining secondary antibodies were removed with PBST, and sections were then stained with DAB (G1212-200 T, Servicebio, China). Counterstaining with hematoxylin was performed before differentiation in 1% hydrochloric acid alcohol. Following a series of gradient dehydration steps, sections were mounted using neutral gum. Digital imaging of the sections was performed with a Pannoramic MIDIII digital microscope (3D HISTECH Ltd., Budapest, Hungary) to evaluate mitophagy and inflammatory infiltration.

### TUNEL assay

A TMR TUNEL Cell Apoptosis Detection Kit (Servicebio, Wuhan, China, G1502) was employed in accordance with the manufacturer's guidelines to assess cell apoptosis in mouse hippocampal tissues. Initially, the sections of mouse hippocampal tissue were deparaffinized. This was followed by pretreatment with 0.1 M sodium citrate (pH 6.0) at 65 °C for 30 min, after which the sections were incubated with the TUNEL reaction mixture at 37 °C within a moist and darkened chamber for 1 h. Apoptotic cells were identified using a confocal microscope (Leica, Germany). For quantitative analysis, ten fields of view were randomly chosen from each section, and the density of TUNEL-positive cells per square millimeter was determined.

### Transmission electron microscopy

Mouse hippocampal tissue was preserved using an electron microscopy fixative (Servicebio, G1102) before undergoing a series of standard histopathological steps, including dehydration, osmication, embedding, sectioning, and staining. The ultrastructural details of the hippocampal neurons were then closely examined using a Hitachi H7700 electron microscope.

### Immunofluorescence

Primary hippocampal neurons, isolated from embryonic rats at gestational days 16 to 18, were cultured on glass coverslips for a duration of 14 days. These cells were then exposed to CSF from patients, both with and without anti-NMDAR antibodies (at a concentration of 4 µg/ml), for a period of 24 h. Concurrently, an equivalent concentration of ART (4 µg/ml) or PBS was administered to the cells. Following this treatment, the cells were rinsed thrice with PBS, fixed in 4% paraformaldehyde for 20 min, permeabilized using 0.1% Triton X-100 in PBS (GC204003, Servicebio) for 5 min, and subsequently blocked with 2% bovine serum albumin (BSA, N0008-100, ZOSEN) in PBS for an hour. The nuclei of the cells were stained with DAPI. The primary antibodies employed targeted GluN1 (114003, Synaptic Systems), MAP2 (M4403, Sigma-Aldrich, Germany), and ds-DNA (sc-58749, Santa Cruz, USA). The secondary antibodies used included goat anti-rabbit IgG H&L (Alexa Fluor® 488, Abcam, ab150077), goat anti-rabbit IgG H&L (Alexa Fluor® 594, Abcam, ab150080), and goat anti-mouse IgG H&L (Alexa Fluor® 647, Abcam, ab150115). Imaging of the coverslips was performed with a confocal microscope (Leica, Germany). Mouse brain tissue was fixed overnight with 4% paraformaldehyde, followed by cardiac perfusion with precooled PBS and 4% paraformaldehyde. This was succeeded by dehydration using sucrose solutions (20% and 30%) at 4 °C. Post-dehydration, the brain tissue was embedded in optimal cutting temperature compound (OCT, SAKURA, 4583) and sectioned into 5-μm slices. These sections were permeabilized with 0.1% Triton X-100 in PBS (Servicebio, GC204003) for 15 min and blocked with 2% BSA (ZOSEN, N0008-100) in PBS for an hour. The sections were incubated overnight at 4 °C with the primary antibody, followed by incubation with the secondary antibody at room temperature for an hour the next day. The primary antibodies targeted GluN1 (Synaptic Systems, 114003), PINK1 (Proteintech, 23274–1-AP), Parkin (Santa Cruz, sc-32282), and MAP2 (Sigma-Aldrich, M4403). The secondary antibodies employed were goat anti-rabbit IgG H&L (Alexa Fluor® 488, Abcam, ab150077), goat anti-rabbit IgG H&L (Alexa Fluor® 594, Abcam, ab150080), and goat anti-rabbit IgG H&L (Alexa Fluor® 647, Abcam, ab150115). The sections were also imaged using a confocal microscope (Leica, Germany), facilitating detailed observation of the specimens.

### Western blot analysis

Mouse hippocampal tissue was lysed using a denaturing cell lysis buffer (Invent, SD-001) supplemented with a protease inhibitor (Fdbio Science, FD1001) and a phosphatase inhibitor (Fdbio Science, FD1002). The protein concentration was quantified using a bicinchoninic acid (BCA) assay kit (Fdbio Science, FD2001). Subsequently, fifty micrograms of protein per sample were resolved on an SDS–polyacrylamide gel for electrophoresis and then electroblotted onto a polyvinylidene fluoride (PVDF) membrane. The membrane was blocked with 5% bovine serum albumin (BSA, ZOSEN, N0008-100) at room temperature for one hour, followed by incubation with the primary antibody at 4 °C overnight. This was succeeded by incubation with the appropriate secondary antibody. Detection of protein bands was accomplished using an Odyssey imaging system (LI-COR, USA), and the densities of the protein bands were quantitatively analyzed using ImageJ software (National Institutes of Health, Bethesda, MD, USA).

### AAV vector packaging

AAV-PINK1 shRNA (targeting mouse *Pink1*) and AAV sh-vector were sourced from OBIO Chemical Technology Co., Ltd. (Shanghai, China). The company conducted in-house validation of these products, quantifying the titers of AAV-PINK1 shRNA and AAV vector using quantitative PCR (qPCR). This analysis revealed titers of 2.6 × 10^12^ and 7.41 × 10^12^ genome copies per milliliter, respectively. The specific sequence for PINK1 shRNA utilized in this study was as follows: CCTGGCTGACTATCCTGATAT.

### In vivo silencing of PINK1 expression

C57BL/6 mice were systematically allocated into four distinct groups: the sham + PBS + sh-vector group, PTM + PBS + sh-vector group, PTM + ART + sh-vector group, and PTM + PBS + sh-PINK1 group. Seven days before undergoing brain surgery, mice assigned to the PTM + PBS + sh-PINK1 group received stereotaxic injections of 200 nl of viral vector, encapsulating 2.6 × 10^12^ genome particles, aimed at the bilateral hippocampal CA1 region (coordinates: AP: -2.00 mm, ML: ± 1.00 mm, DV: -2.6 mm) through a precision microsyringe. The remaining groups were administered a comparable volume of AAV vector lacking genome particles, following the identical procedure. Post-operatively, mice in the PTM + ART + sh-vector and PTM + PBS + sh-PINK1 shRNA groups were treated with ART (100 mg/kg, administered intraperitoneally twice daily), whereas mice in the sham + PBS + sh-vector and PTM + PBS + sh-vector groups received a corresponding volume of PBS. Three weeks subsequent to the treatment, the efficacy of PINK1 protein suppression in the hippocampus was confirmed through Western blot analysis and immunofluorescence techniques.

### AAV transfection in vitro

SH-SY5Y cells were stratified into four experimental cohorts: CSF IgG(-) + PBS + vector, CSF IgG( +) + PBS + vector, CSF IgG( +) + ART + vector, and CSF IgG( +) + ART + sh-PINK1. Initially, the cells were cultured in 6-well plates until reaching a confluency of 30%–40% in preparation for transfection. Subsequently, 19 µl of a viral vector, encapsulating 2.6 × 10^12^ genome particles, was administered to the CSF IgG( +) + ART + sh-PINK1 group. In contrast, the other three cell groups were treated with an equivalent volume of the AAV empty vector. Forty-eight hours later, the culture medium was replaced with reduced-serum Opti-MEM. Following this medium change, the cells were exposed to CSF from patients, both with and without anti-NMDAR antibodies (at a concentration of 8 µg/ml), and concurrently treated with either ART (1 µg/ml) or a matching volume of PBS for 24 h. The effectiveness of PINK1 protein reduction in the cells was subsequently confirmed via Western blot analysis.

### Administration of reagent

ART, sourced from Guilin Pharmaceutical Corporation in China, was formulated into a 10 mg/ml stock solution using sodium bicarbonate (NaHCO_3_) and saline. This solution was then diluted to the requisite concentration with either saline or cell culture medium, tailored to the specific requirements of the experimental setup.

### Observation of mitochondrial morphology and detection of mitochondrial membrane potential and mitochondrial ROS

Primary hippocampal neurons were labeled with MitoTracker Deep Red (Thermo Fisher Scientific, M22426) to visualize mitochondrial morphology via a confocal microscope (Leica, Germany). Following the manufacturer's guidelines, cells were rinsed with PBS and then incubated with the TMRM probe (Invitrogen, I34361) in a 37 °C/5% CO_2_ environment shielded from light for 30 min. Subsequent to probe removal with PBS, images were captured using a confocal microscope (Leica, Germany). The alterations in mitochondrial membrane potential were assessed based on the intensity of red or green fluorescence. For quantitative analysis, ImageJ software (NIH, Bethesda, MD, USA) was employed.

### Statistical analysis

In this investigation, qualitative data are derived from a minimum of three independent experiments, and quantitative data are expressed as mean ± SD. *T*-tests were utilized to assess the statistical significance of differences between two distinct groups. For evaluating disparities among multiple groups, one-way analysis of variance (ANOVA) was applied. Post hoc comparisons for datasets with homogenous variances were made using the Bonferroni correction, whereas for datasets with heterogeneous variances, Dunnett's C test was implemented. All statistical analyses were conducted using GraphPad Prism software, with a *P* value of less than 0.05 deemed significant for indicating statistical relevance.

## Results

### The PTM induced by IgG-positive CSF leads to elevated neuronal apoptosis and insufficient mitophagy

**Fig. 1:**
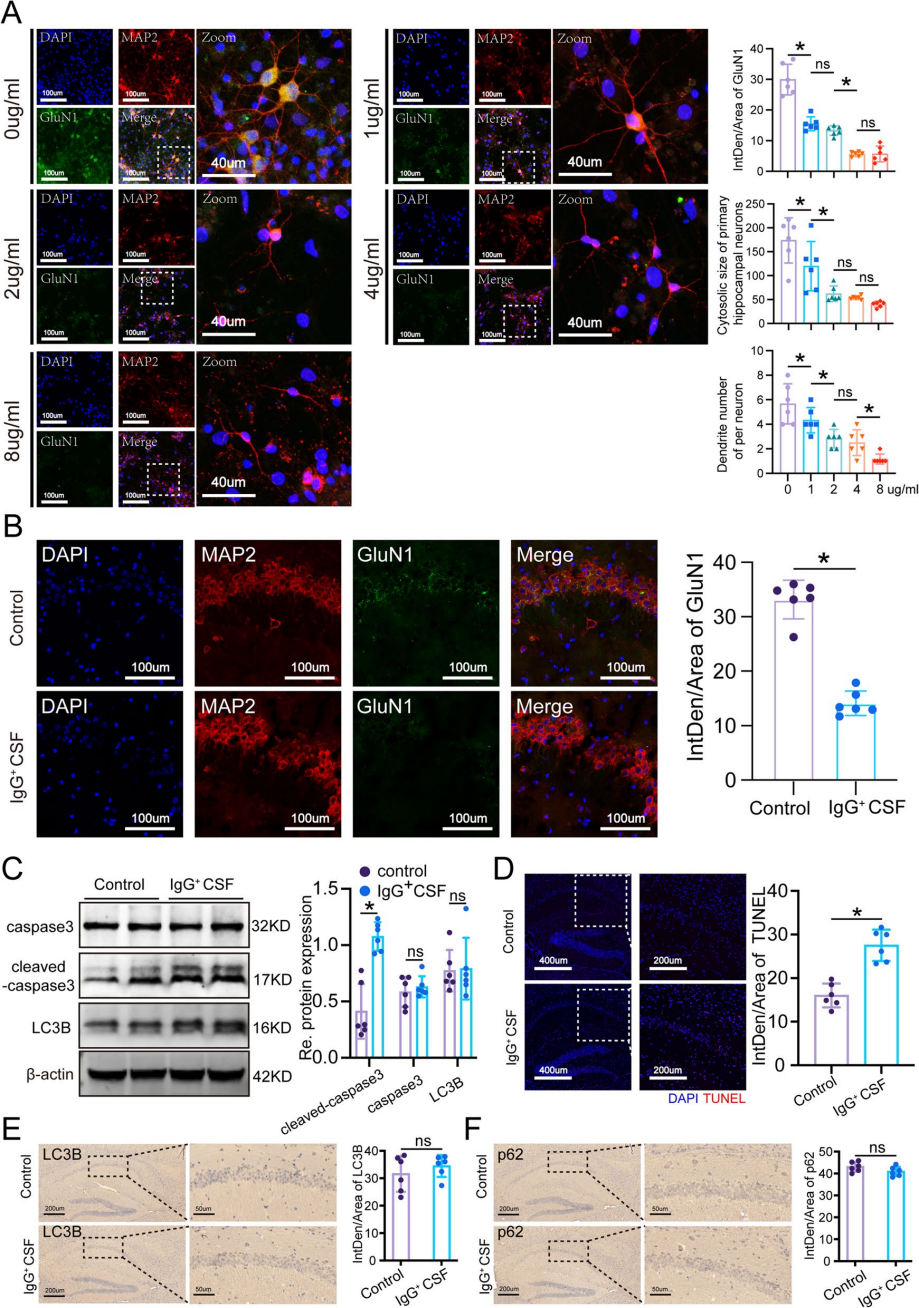
The passive transport model induced by IgG-positive cerebrospinal fluid exhibits elevated neuronal apoptosis and insufficient mitophagy. **A** Typical immunofluorescence images of primary hippocampal neurons treated with IgG-positive CSF at different concentrations and quantitative statistical analysis of GluN1 receptor levels. DAPI represents the cell nucleus (blue), MAP2 represents microtubule-associated protein 2 in neurons (red), and GluN1 represents the GluN1 receptor in hippocampal neurons (green). **B** Typical immunofluorescence images of hippocampal tissue from mice passively immunized with IgG-positive CSF and control mice, as well as quantitative statistical analysis of GluN1 receptor levels. DAPI represents the cell nucleus (blue), MAP2 represents microtubule-associated protein 2 in neurons (red), and GluN1 represents the GluN1 receptor in hippocampal neurons (green). **C** Western blot analysis of hippocampal tissue from mice passively immunized with IgG-positive CSF and control mice was performed to detect the protein expression levels of cleaved caspase3, caspase3, and LC3B, as well as to perform quantitative statistical analysis of the corresponding proteins. **D** TUNEL immunofluorescence staining of hippocampal tissue from mice passively immunized with IgG-positive CSF and control mice and quantitative statistical analysis of the TUNEL staining data. DAPI represents the cell nucleus (blue), and TUNEL represents apoptotic cells (red). **E** Typical immunohistochemical images of LC3B in the hippocampal tissue of mice passively immunized with IgG-positive CSF and control mice, along with quantitative statistical analysis. **F** Typical immunohistochemical images of p62 in the hippocampal tissue of mice passively immunized with IgG-positive CSF and control mice, along with quantitative statistical analysis. *n* = 6, *P < 0.05 versus the indicated group

In our in vitro experiments, primary hippocampal neurons cultured from embryonic rats were exposed to varying concentrations of CSF obtained from patients with anti-NMDAR encephalitis or control subjects. We observed a dose-dependent increase in the internalization of the GluN1 receptor with escalating antibody concentrations (Fig. 1A). This trend reached a plateau at 4 µg/ml, prompting us to select this concentration for subsequent cellular studies. Moving to our in vivo investigations, C57BL/6 J mice were subjected to intracerebroventricular infusion of pooled CSF containing anti-NMDAR antibodies or control CSF. Immunofluorescence staining confirmed a reduction in GluN1 signal intensity in mice receiving CSF from patients, thereby establishing the passive transfer model (PTM) of anti-NMDAR encephalitis (Fig. 1B). Western blot analysis of hippocampal tissue from PTM mice revealed an intriguing pattern: an increase in cleaved caspase-3 levels accompanied by a decrease in total caspase-3 protein levels compared to controls (Fig. 1C). This observation suggests a potential shift in caspase-3 processing within the hippocampus under conditions of anti-NMDAR encephalitis. However, levels of LC3B, a key marker of autophagic activity, remained consistent across both PTM and control groups (Fig. 1C), indicating no significant alteration in baseline autophagy levels. Furthermore, TUNEL assays provided compelling evidence of heightened neuronal apoptosis in the hippocampus of PTM mice relative to controls (Fig. 1D). This finding underscores the deleterious impact of anti-NMDAR encephalitis on neuronal viability and survival within the central nervous system. Immunohistochemical analyses did not uncover substantial differences in LC3B and p62 protein expression between PTM and control groups (Fig. 1E and F), implying a muted activation of mitophagy in the PTM condition. This observation raises intriguing questions about the interplay between mitochondrial quality control mechanisms and the pathogenesis of anti-NMDAR encephalitis, warranting further investigation into the molecular underpinnings of mitophagy dysregulation in this disease context. In summary, our comprehensive approach utilizing both in vitro and in vivo models sheds light on the interplay between neuronal apoptosis, mitochondrial dysfunction, and mitophagic processes in the pathogenesis of anti-NMDAR encephalitis.

### Mitochondrial damage occurs in primary hippocampal neurons treated with anti-NMDAR antibodies, and ART can alleviate this damage

**Fig. 2:**
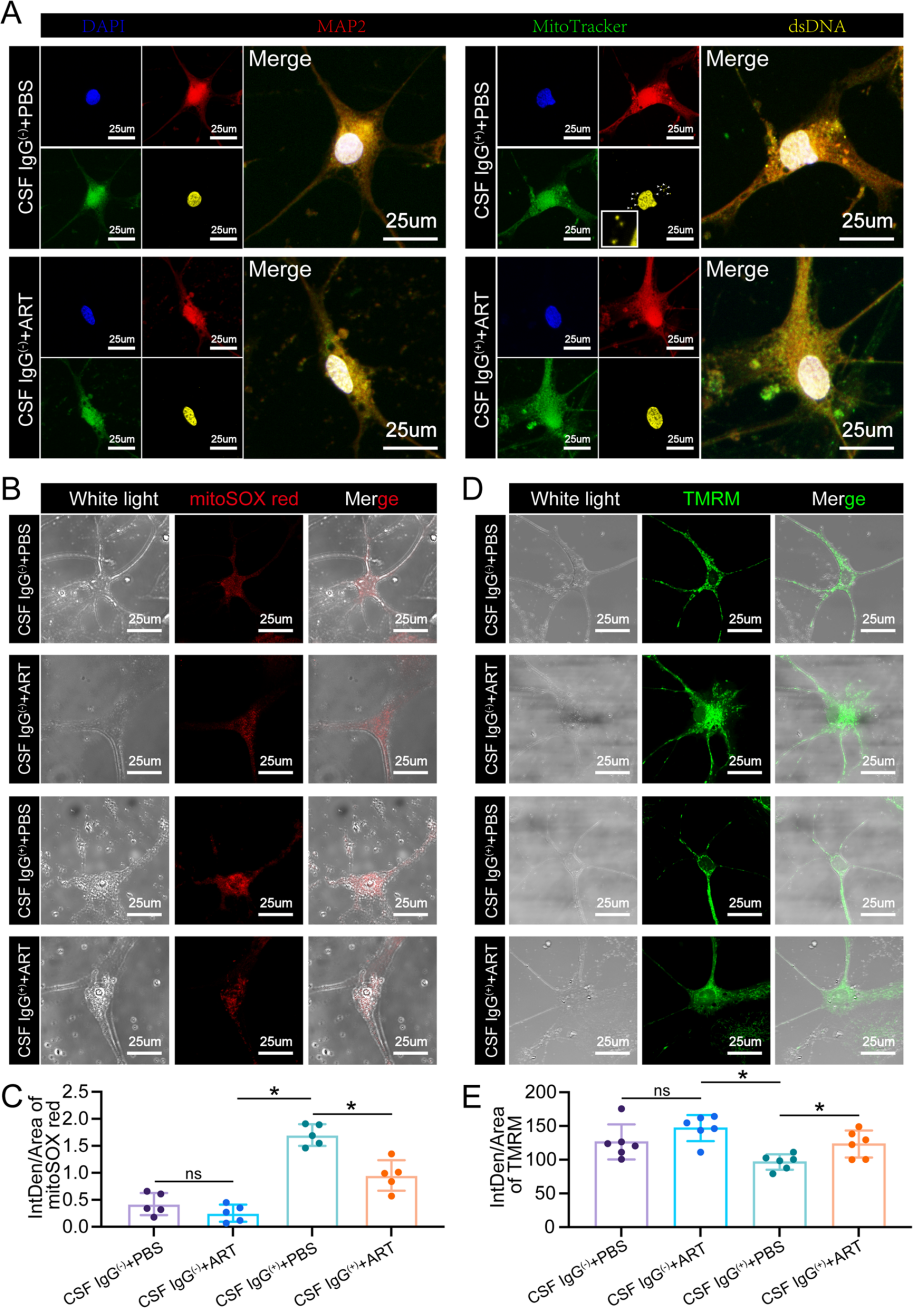
ART inhibits IgG-positive cerebrospinal fluid-induced mitochondrial dysfunction in vitro. **A** Immunofluorescence staining of hippocampal neurons from the different groups was performed to evaluate whether ART can inhibit mitochondrial damage caused by PTM. DAPI represents the cell nucleus (blue), MAP2 represents microtubule-associated protein 2 (red), MitoTracker represents mitochondria labeled with the probe MitoTracker, dsDNA represents double-stranded DNA, and scattered fluorescence signals around the cell nucleus represent mtDNA. **B** Immunofluorescence staining of hippocampal neurons in different groups to label the mitochondrial superoxide complex. The white light represents the bright field (gray), and the red represents a fluorescence probe that selectively detects superoxide species within mitochondria (red), with a lighter red color indicating more severe mitochondrial dysfunction. **C** Quantitative statistical analysis of the mitoSOX red fluorescence signal. *n *= 5, *P < 0.05 versus the indicated group. **D** Immunofluorescence staining with TMRM was performed to evaluate the mitochondrial membrane potential in different groups of hippocampal neurons. The white light represents the bright field (gray), TMRM represents the mitochondrial membrane potential (green); once the mitochondrial membrane potential is lost, TMRM no longer accumulates, resulting in a weakened or absent fluorescence signal. **E** Quantitative statistical analysis of the TMRM fluorescence signal. CSF(IgG^−^) represents cerebrospinal fluid given without anti-NMDAR antibodies to the normal control group, CSF(IgG^+^) represents cerebrospinal fluid given with anti-NMDAR antibodies to the experimental group, and ART represents artemisinin. *n* = 6, *P < 0.05 versus the indicated group

Initially, we explored mitochondrial damage in primary hippocampal neurons. These neurons were cultured in neurobasal medium enriched with B27, 2 mM GlutaMAX, and 100 U/mL penicillin/streptomycin for a duration of 14 days, followed by a 24-h treatment with 4 µg/ml ART. Immunofluorescence analysis, employing anti-dsDNA antibodies, highlighted intact mtDNA structures in the CSF IgG( +) + PBS group (Fig. 2A). Notably, primary hippocampal neurons subjected to anti-NMDAR antibodies from patients displayed a significant translocation of dsDNA to the cytoplasm, with instances of dsDNA colocalization with mitochondria, in contrast to the control group. However, post-ART treatment, minimal mtDNA leakage was observed in the neurons (Fig. 2A). Utilizing MitoSOX Red and TMRM for mitochondrial labeling, we found that neurons treated with CSF containing anti-NMDAR antibodies exhibited pronounced signs of mitochondrial damage and reduced mitochondrial membrane potential. In contrast, ART treatment appeared to enhance the functionality of damaged mitochondria (Fig. 2B-E). These findings suggest that ART plays a crucial role in alleviating mitochondrial damage.

### ART activates the PINK1/PARKIN pathway, increased mitophagy and reduced neuronal apoptosis in mice treated with anti-NMDAR antibodies

**Fig. 3:**
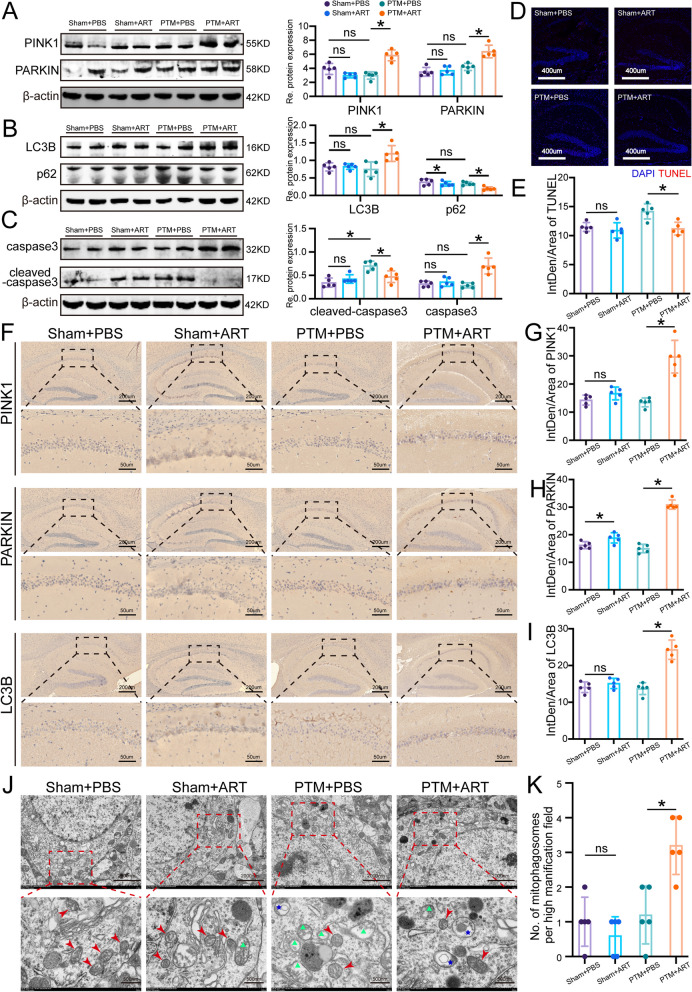
ART upregulates PINK1/PARKIN activity, suppresses neuronal apoptosis and enhances mitophagy in vivo*.* **A** Western blot analysis of hippocampal tissue from mice for PINK1 and PARKIN proteins in the different groups and corresponding quantitative statistical graphs. **B** Western blot analysis of hippocampal tissue from mice for LC3B and p62 proteins in the different groups and corresponding quantitative statistical graphs. **C** Western blot analysis of cleaved caspase-3 and caspase-3 proteins in the different groups and corresponding quantitative statistical graphs. **D** Immunofluorescence staining of TUNEL-stained hippocampal tissues from the different groups. DAPI represents the cell nucleus (blue), and TUNEL represents apoptotic neurons. **E** Quantitative statistical graph corresponding to panel D. **F** Representative immunohistochemical images of PINK1, PARKIN, and LC3B proteins in the different groups. **G-I** Quantitative statistical graphs of PINK1, PARKIN, and LC3B protein expression corresponding to panel F.** J** Representative transmission electron microscopy images of hippocampal tissue from the different groups. Red arrows indicate normal mitochondria, green triangles indicate mitochondria with damage and swelling, and blue asterisks indicate mitochondrial autophagosomes. **K** Quantitative statistical graph corresponding to panel J indicating the number of autophagosomes. Sham represents the sham surgery group, PTM represents the passive transfer model, and ART represents artemisinin. *n* = 5, *P < 0.05 versus the indicated group

To comprehensively evaluate the impact of ART on anti-NMDAR encephalitis PTM, we conducted extensive in vivo investigations. Our Western blot analyses of hippocampal tissue from mice yielded profound insights. Notably, PTM mice treated with ART, administered at a dosage of 100 mg/kg twice daily for 14 days, exhibited significant upregulation of key proteins in the PINK1/PARKIN pathway, including PINK1, PARKIN, and LC3B. Conversely, the expression of p62, a marker of impaired autophagy, was markedly reduced in the ART-treated group compared to the untreated PTM group. Moreover, a compelling increase in Caspase 3 and a decrease in cleaved Caspase 3 expression were observed, indicative of ART's potential in modulating apoptotic pathways (Fig. 3A-C). Further validation through TUNEL staining demonstrated a substantial reduction in neuronal apoptosis within the hippocampal tissue of the PTM + ART group compared to the PTM + PBS group (Fig. 3D, E). Immunohistochemical analysis provided additional insights into the cellular mechanisms underlying ART's neuroprotective effects. Specifically, enhanced expression levels of PINK1, PARKIN, and LC3B were observed in the CA1 region of the hippocampus in PTM mice following ART treatment, suggesting an augmentation of mitophagy processes in response to ART administration (Fig. 3F-I). These findings collectively indicate that ART possesses the capability to enhance the activity of the PINK1/PARKIN pathway, thereby promoting autophagic clearance of damaged mitochondria and mitigating neuronal apoptosis in the PTM model. Further corroborating our results, transmission electron microscopy (TEM) observations revealed a striking reduction in the incidence of mitochondria exhibiting swelling-induced damage and an increased presence of mitochondrial autophagosomes in the PTM + ART group compared to the PTM + PBS group (Fig. 3J and K). This comprehensive characterization underscores the potential of ART as a promising therapeutic intervention for mitigating mitochondrial dysfunction and neuronal loss in anti-NMDAR encephalitis.

### ART improved the behavioral performance of PTM mice treated with anti-NMDAR antibodies

**Fig. 4:**
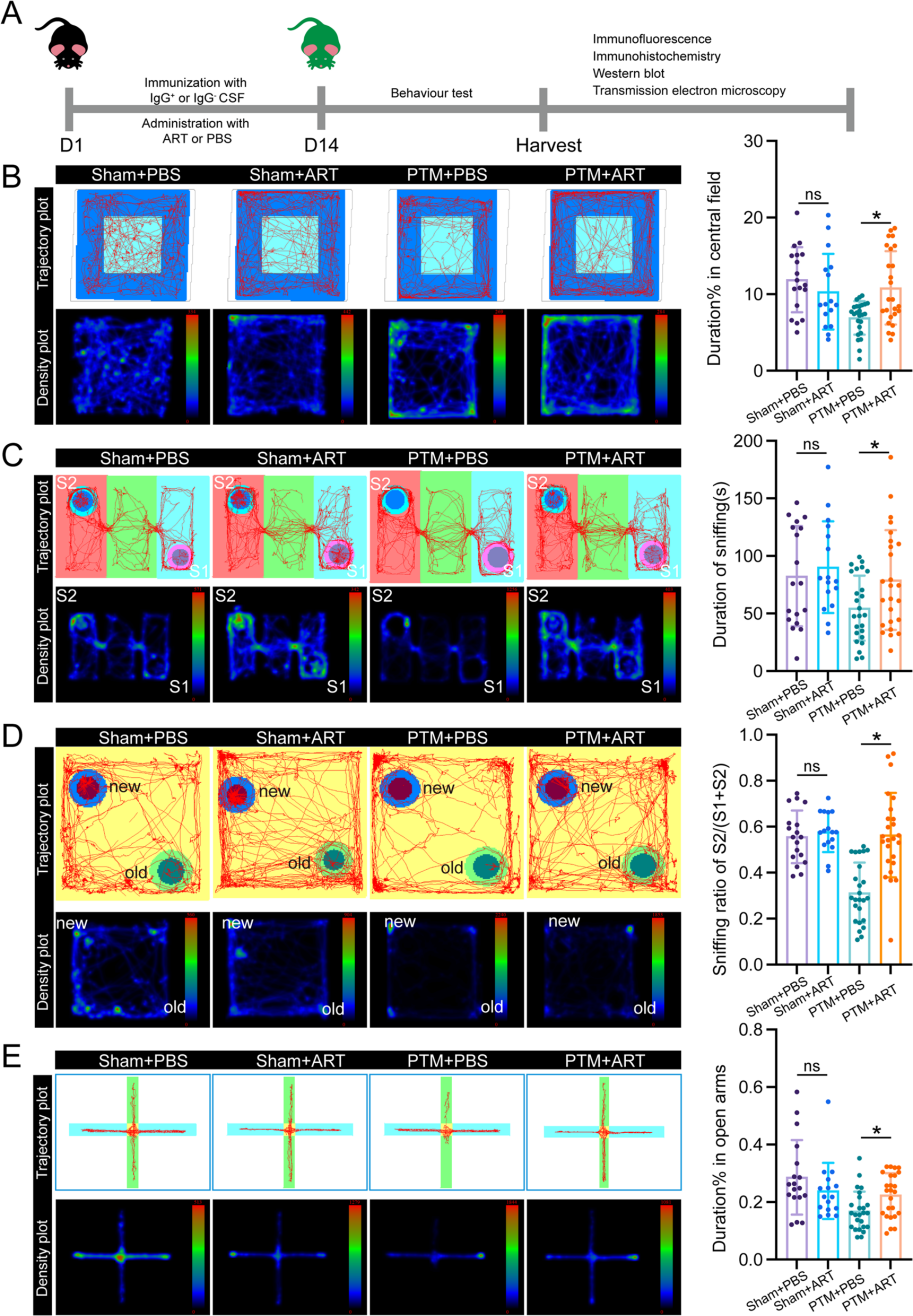
ART improves behavioral parameters in IgG-positive cerebrospinal fluid-induced passive transport model mice. **A** Diagram of the experimental procedure. **B** Trajectory and density plots of the different groups of mice in the open field test, along with corresponding quantitative statistical graphs. *n* = 18, 16, 25, and 25 for the sham + PBS, sham + ART, PTM + PBS and PTM + ART groups, respectively. More tracks or a higher density in the central area indicate stronger spontaneous locomotor activity and more active exploratory behavior toward new environments in the mice. **C** Trajectory and density plots of the three-chamber social interaction experiments in different groups are shown, along with corresponding quantitative statistical graphs. *n* = 18, 16, 25, and 25 for the sham + PBS, sham + ART, PTM + PBS and PTM + ART groups, respectively. S1 refers to the cup containing stranger mouse #1; S2 refers to the cup containing stranger mouse #2. **D** Trajectory and density plots illustrating the novel object recognition experiments in different groups, along with corresponding quantitative statistical graphs. *n* = 18, 16, 25, and 25 for the sham + PBS, sham + ART, PTM + PBS and PTM + ART groups, respectively. new represents the new object; old represents the old object. **E** Trajectory and density plots of the different groups of mice in the elevated plus maze test, along with corresponding quantitative statistical graphs. *n* = 18, 16, 25, and 25 for the sham + PBS, sham + ART, PTM + PBS and PTM + ART groups, respectively. The horizontal arms represent the enclosed arms, while the vertical arms represent the open arms. Sham represents the sham surgery group, PTM represents the passive transfer model, and ART represents artemisinin. *P < 0.05 versus the indicated group

To comprehensively evaluate the neurological functions of the mice, we meticulously implemented a battery of behavioral tests following the pre-established study protocol (Fig. 4A). These tests were thoughtfully selected to encompass various aspects of behavior and cognitive function, including exploratory behavior, social interaction, recognition memory, and anxiety-like behavior. In the open field test (OFT), mice from the PTM + PBS group exhibited a pronounced preference for peripheral movement over exploring the central area, indicative of heightened anxiety-like behavior, whereas mice treated with ART (PTM + ART group) displayed a more balanced exploration pattern, suggesting reduced anxiety (Fig. 4B). During the three-chamber test (TCT), a widely used paradigm to assess social behavior, we observed that mice in the PTM + PBS group showed decreased interaction time with a stranger mouse compared to those in the PTM + ART group, indicating improved social interaction following ART treatment (Fig. 4C). In the novel object recognition test (NORT), mice in the PTM + ART group demonstrated a higher recognition index than those in the PTM + PBS group, reflecting enhanced recognition memory. This suggests that ART administration may ameliorate cognitive deficits associated with anti-NMDAR encephalitis (Fig. 4D). Furthermore, in the elevated plus maze (EPM) test, commonly used to evaluate anxiety-like behavior, mice in the PTM + PBS group spent less time exploring the open arms compared to those in the PTM + ART group, indicating reduced anxiety levels following ART treatment (Fig. 4E). Taken together, these comprehensive behavioral assessments reveal that mice treated with ART exhibit improvements in anxiety-like behavior, social interaction deficits, recognition memory impairment, and cognitive deficits associated with anti-NMDAR encephalitis. These findings underscore the potential of ART as a therapeutic intervention to enhance the behavioral outcomes of mice undergoing NMDAR antibody therapy in the context of anti-NMDAR encephalitis.

### ART activates mitophagy through the PINK1/PARKIN pathway to exert neuroprotective effects on PTM mice treated with anti-NMDAR antibodies

**Fig. 5:**
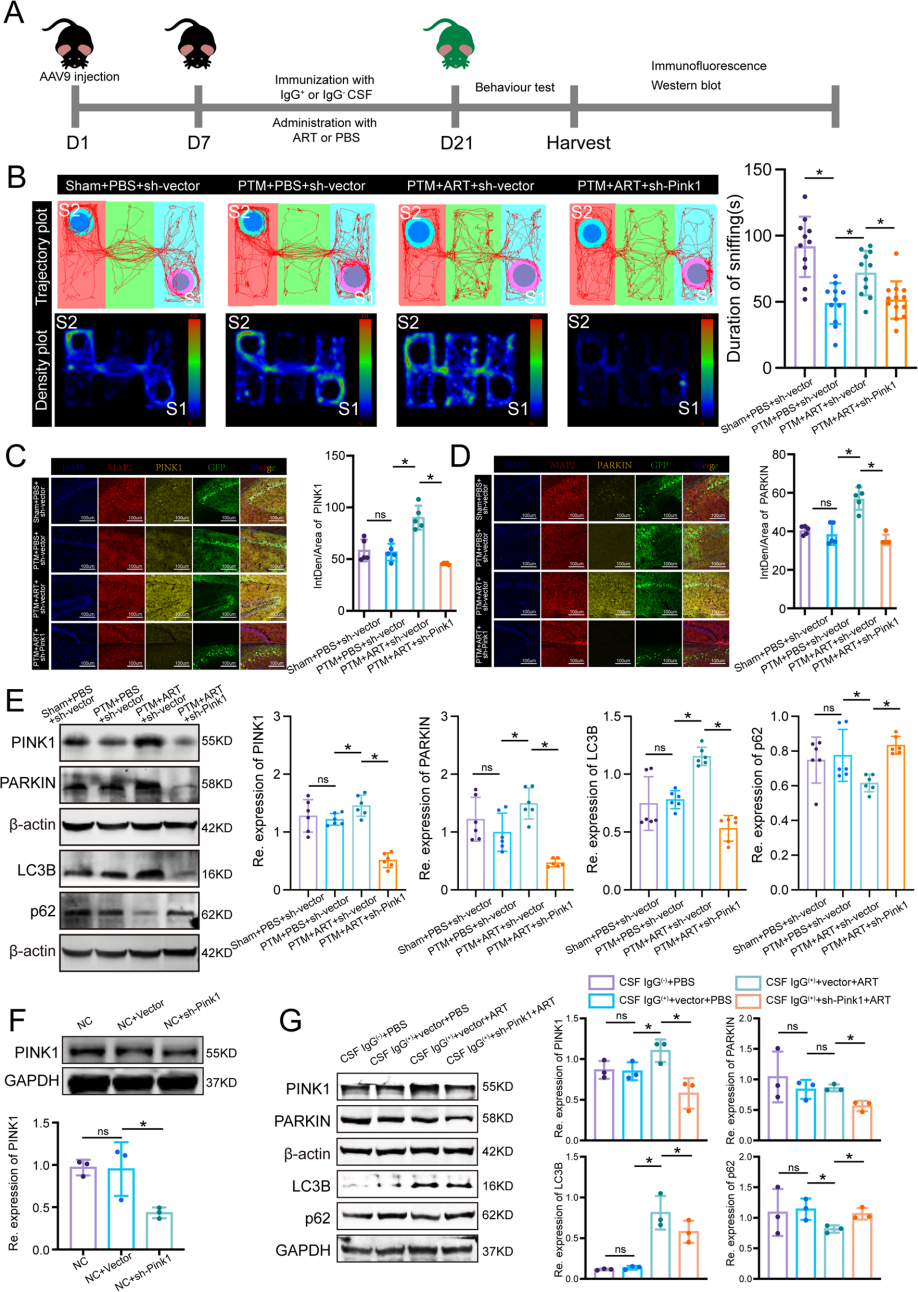
ART promotes mitochondrial autophagy and ameliorates behavioral abnormalities induced by IgG-positive cerebrospinal fluid by enhancing the PINK1/PARKIN signaling pathway. **A** Experimental schedule: The AAV9 vector was injected into the hippocampal tissue on D1. On D7, a passive transport model was established through the placement of a microinfusion pump, along with the intraperitoneal injection of ART. Behavioral testing was conducted on D21. **B** Trajectory and density plots illustrate the three-chamber social interaction experiments in different groups, along with corresponding quantitative statistical graphs. *n* = 11, 11, 11, and 13 for the sham + PBS, sham + ART, PTM + PBS and PTM + ART groups, respectively. S1 refers to the cup containing stranger mouse #1, while S2 refers to the cup containing stranger mouse #2. **C** Immunofluorescence staining of hippocampal tissue from the different groups and quantitative statistical analysis of PINK1 fluorescence signals. *n* = 6. DAPI represents cell nuclei (blue), MAP2 represents microtubule-associated protein 2 (red), PINK1 represents the PINK1 protein in the hippocampal tissue labeled with anti-PINK1 antibody (yellow), and GFP represents hippocampal neurons infected with GFP-labeled virus (green). **D** Immunofluorescence staining of hippocampal tissue from the different groups and quantitative statistical analysis of PARKIN fluorescence signals. *n* = 6. DAPI represents the cell nuclei (blue), MAP2 represents microtubule-associated protein 2 (red), PARKIN represents the PARKIN protein in the hippocampal tissue labeled with the anti-PARKIN antibody (yellow), and GFP represents hippocampal neurons infected with the GFP-labeled virus (green). **E** Western blot analysis of the relative protein expression levels of PINK1, PARKIN, LC3B, and p62 in dissociated hippocampal tissue from the different groups, along with corresponding quantitative statistical graphs. *n* = 6. **F** Western blot analysis and quantitative analysis of *Pink1* knockdown after AAV9 transfection in the SH-SY5Y cell line; *n* = 3. **G** Relative expression levels of the PINK1, PARKIN, LC3B, and p62 proteins in different groups of SH-SY5Y cells were detected via Western blotting, and the proteins were quantified; *n* = 3. CSF(IgG^−^) represents cerebrospinal fluid without anti-NMDAR antibodies that was administered to the normal control group, and CSF(IgG^+^) represents cerebrospinal fluid with anti-NMDAR antibodies that was administered to the experimental group. Sham represents the sham surgery group, PTM represents the passive transport model, and ART represents artemisinin. sh-vector represents the empty vector, while sh-PINK1 represents the AAV9 vector capable of downregulating PINK1 expression. *P < 0.05 versus the indicated group

To explore ART's potential to initiate mitophagy through the PINK1/PARKIN pathway, we conducted an experimental setup (Fig. 5A), commenced with the bilateral hippocampal CA1 region injection of a viral vector containing shRNA, using a microinjector to deliver a precise volume of 200 nl in vivo. On the seventh day post-injection, we established a transfection-based mouse model by administering ART intraperitoneally. After a three-week duration, we engaged in a comprehensive evaluation involving behavioral testing, immunofluorescence staining, and western blot analysis. Brain imaging confirmed the successful transfection in the hippocampal region, as evidenced by staining (Fig. SF1A). Additional immunofluorescence staining of the tissue highlighted a significant reduction in GluN1 fluorescence intensity in the PTM model, indicating effective model creation. The detection of GFP fluorescence further validated the efficiency of the transfection process (Fig. SF1B). Behavioral analyses revealed that mice with *Pink1* knockdown, upon ART treatment, exhibited intensified anxiety, depressive behavior, and social disturbances in the PTM + PBS + sh-vector group compared to the sham + PBS + sh-vector group. Remarkably, ART administration mitigated these behaviors (Fig. 5B, Fig. SF1C and D). This behavior modification by ART was found to be reliant on the PINK1/PARKIN pathway's activation. Immunofluorescence staining results demonstrated a marked increase in PINK1 and PARKIN fluorescence signals in the PTM + ART + sh-vector group compared to the PTM + PBS + sh-vector group, suggesting that ART upregulates the expression of PINK1 and PARKIN, thereby activating the PINK1/PARKIN signaling pathway (Fig. 5C and D). Western blot findings revealed elevated levels of PINK1, PARKIN, and LC3B in the hippocampal tissue of the PTM + ART + sh-vector group compared to the PTM + ART + sh-PINK1 group, where p62 expression was also higher (Fig. 5E). In the SH-SY5Y cell line, effective *Pink1* knockdown was confirmed through AAV9-sh-Pink1 transfection (Fig. 5F). Further, in vitro experiments in the anti-NMDAR encephalitis cell model showed that IgG-positive CSF exposure led to a significant downregulation of PINK1, PARKIN, and LC3B, indicating a deficiency in mitophagy. However, ART administration was found to enhance mitophagy, an effect that could be inhibited by *Pink1* knockdown, underscoring that ART augments autophagy by activating the PINK1/PARKIN pathway (Fig. 5G). In summary, our findings suggest that ART enhances mitophagy through the PINK1/PARKIN pathway and exerts neuroprotective effects in PTM mice undergoing treatment.

## Discussion

In this study, we aimed to evaluate the feasibility and investigate the underlying mechanism of ART for treating anti-NMDAR encephalitis through both in vitro and in vivo experiments. We hypothesized that in the context of anti-NMDAR encephalitis, there exists mitophagy and neuronal apoptosis, however, mitophagy is insufficient. ART could activate the PINK1/PARKIN pathway to stimulate mitophagy, mitigate mitochondrial damage, reduce neuronal apoptosis, and exert neuroprotective effects.

We successfully explored the conditions of mitochondrial damage, mitophagy, and neuronal apoptosis in anti-NMDAR encephalitis. Our findings suggest that, compared to the control group, neurons treated with anti-NMDAR antibodies showed significant increases in mitochondrial damage indicators such as mitoSOX and mtDNA, along with a decrease in mitochondrial membrane potential (TMRM), indicating increased mitochondrial damage. Markers of mitophagy, including PINK1, PARKIN, and LC3B, did not show a significant increase, whereas indicators of neuronal apoptosis were notably higher in comparison to the negative control group. In the PTM of anti-NMDAR encephalitis mice treated with ART, behavioral studies suggested that ART could improve the behavioral performance of model mice. Indicators of mitochondrial damage were alleviated, markers of mitophagy increased, and the number of autophagic bodies in mitochondria was higher compared to the disease group as observed under TEM. TUNEL assay indicated a reduction in neuronal apoptosis in the hippocampus, consistent with our previous clinical research outcomes [32, 34]. In vitro and in vivo rescue experiments showed that the neuroprotective effect of ART disappeared after knockdown of *Pink1*, suggesting that ART exerts its neuroprotective role through the activation of mitophagy via the PINK1/PARKIN pathway. These results imply that mitochondrial damage and insufficient mitophagy may be one of the pathogenic mechanisms of anti-NMDAR encephalitis, and modulating mitophagy could become a potential target for treatment. This represents an important complement to the understanding of the pathogenesis and treatment strategies for anti-NMDAR encephalitis and provides a new direction for research into the pathogenesis of central nervous system immune-related diseases, even other central nervous system diseases. It can also be anticipated that research progress in mitophagy signaling will play a significant role in developing new precise medical treatment strategies. Given the high efficacy and safety of ART, which is a first-line drug for cerebral malaria and severe malaria and can cross the blood–brain barrier, ART emerges as a promising potential treatment for anti-NMDAR encephalitis. Further validation through larger scale randomized controlled trials is feasible and has been included in our future research plans.

Mitophagy, which mediates the elimination of mitochondria, plays a pivotal role in various processes such as inflammation, metabolic transformation, and cellular reprogramming [35]. Impaired mitochondria not only lack the capability to produce ATP and other biosynthetic products but also release higher levels of reactive oxygen species (ROS). And mitophagy maintains an optimal state of mitochondria by removing dysfunctional or excessive mitochondria [14]. Research on a variety of neurodegenerative diseases commonly acknowledges a dual role of mitochondria in the development of neuroinflammation. On one hand, damaged mitochondria initiate neuroinflammation in the central nervous system through pathways involving pattern recognition receptor (PRR) signaling and the production of ROS. On the other hand, they also promote a selective autophagic process, known as mitophagy, to perform self-clearance and thus avoid excessive inflammation [8]. Prior to this study, we postulated that mitochondrial damage would trigger mitophagy, which through a negative feedback regulation mechanism negatively regulates neuronal apoptosis. In the context of anti-NMDAR encephalitis, the existence of neuronal apoptosis has been a subject of debate. Our findings indicate the presence of minor neuronal apoptosis. In 2015, Josep Dalmau and colleagues discovered, using TUNEL staining in the CA3 region of hippocampal tissues from a passive transfer mouse model of anti-NMDAR encephalitis, a lack of apoptotic cells [36]. In contrast, Marinos C. Dalakas and his team, in 2019, found that long-term exposure to antibodies led to irreversible neurological deficits caused by excessive neuronal death and consequent brain atrophy as evidenced by MRI imaging [3]. Other studies have shown initial stages of anti-NMDAR encephalitis to exhibit brain biopsy or autopsy reports indicating infiltration of B cells, plasma cells, CD4^+^ T cells, and fewer CD8^+^ T cells, along with microglial activation, IgG deposition, and little if any neuronal loss [37–39]. We conjecture that neuronal apoptosis may be associated with factors such as the duration of antibody intervention, disease progression, and animal strains, and that subsequent investigations will require comprehensive validation from clinical brain biopsies or autopsies, combined with clinical manifestations and imaging data. Furthermore, in vivo animal experiments involving changes in experimental conditions, such as varying animal strains, duration of continuous infusion with anti-NMDAR antibodies, and timing of tissue collection across multiple time points, offer a multi-faceted substantiation approach.

In this study, we demonstrated the potential of ART, a mitophagy enhancer, as a treatment for anti-NMDAR encephalitis through both in vitro and in vivo experiments. The results authenticated that ART upregulated the PINK1/PARKIN-dependent pathway, enhancing mitophagy, while concurrently diminishing mitochondrial damage, alleviating neuroinflammation, and reducing neuronal apoptosis. Effective mitochondrial quality control is known to be crucial for cellular and organismal homeostasis, and numerous clinical drug trials have explored the potential of mitophagy as a therapeutic target. Several potent mitophagy inducers have been identified, offering considerable benefits in extending healthy lifespan and protecting animal models and human neurons, including NAD precursors, Urolithin A (UA), Actinonin, spermidine 62, 63, and FDA-approved drugs such as rapamycin and metformin [40–42]. This study is the first research to report mitochondrial damage within anti-NMDAR encephalitis, yet an insufficiency in mitophagy. Intervention with ART enhanced mitophagy and reduced neuronal apoptosis, confirming ART's neuroprotective effects. ART emerges as a promising potential drug for the treatment of anti-NMDAR encephalitis, warranting further in-depth investigation. Moreover, this provides empirical support for the feasibility of utilizing artesunate as a mitophagy regulator in treating anti-NMDAR encephalitis.

This study has several limitations. Firstly, our focus was primarily on mitochondrial damage and mitophagy in neurons, without examining other neural cells, including microglia, which play roles in neuroinflammation and mitophagy. The transition between the resting and activated states of microglia is crucial for maintaining central nervous system homeostasis [43]. Therefore, future research should focus on the interaction between microglia and neurons in mitophagy and explore the role of microglia in this process. Secondly, our assessment was limited to mitophagy through the PINK1/PARKIN-dependent pathway; however, other pathways might also be involved. Different mitophagy pathways may play varying roles in different diseases, thus, future studies need to evaluate additional mitophagy pathways. Thirdly, our study utilized a passive transfer mouse model and primarily observed the acute phase of anti-NMDAR encephalitis in mice, without assessing the impact of ART intervention during the recovery phase. To evaluate the effects of ART on the prognosis of anti-NMDAR encephalitis, further exploration is essential. Fourthly, regarding the clinical application of ART, determining the accurate timing for medication, dosage, and thoroughly assessing the drug's efficacy, stability, and safety, as well as its impact on disease prognosis compared to current frontline treatments, will be the focus of our next-step clinical research. We plan to conduct a multi-sample randomized controlled double-blind trial to substantiate these aspects.

## Conclusion

In the present study, we discovered that mitochondrial damage during the acute phase of anti-NMDAR encephalitis does not autonomously trigger mitophagy. However, administering ART significantly activated mitophagy by enhancing the PINK1/PARKIN pathway, thereby reducing neuronal apoptosis and ameliorating behavioral symptoms in experimental mice. Furthermore, our findings suggest that mitophagy could play a crucial role in combating anti-NMDAR encephalitis, with ART demonstrating a neuroprotective effect by stimulating mitophagy. This investigation highlights a novel therapeutic target and introduces ART as a promising treatment option for anti-NMDAR encephalitis, underscoring the need for further clinical trials to verify its therapeutic effectiveness.

### Supplementary Information

Supplementary Material 1.

Supplementary Material 2.

## Data Availability

No datasets were generated or analysed during the current study.
