# Supplementary material for: Exploring the neuroprotective role of artesunate in mouse models of anti-NMDAR encephalitis: insights from molecular mechanisms and transmission electron microscopy
Source: Cell Commun Signal. 2024 May 14;22:269. doi: 10.1186/s12964-024-01652-4 (PMC11094908; doi:10.1186/s12964-024-01652-4)
Supplement: Supplementary file 1 — Supplementary Material 1. [file 12964_2024_1652_MOESM1_ESM.docx]

**
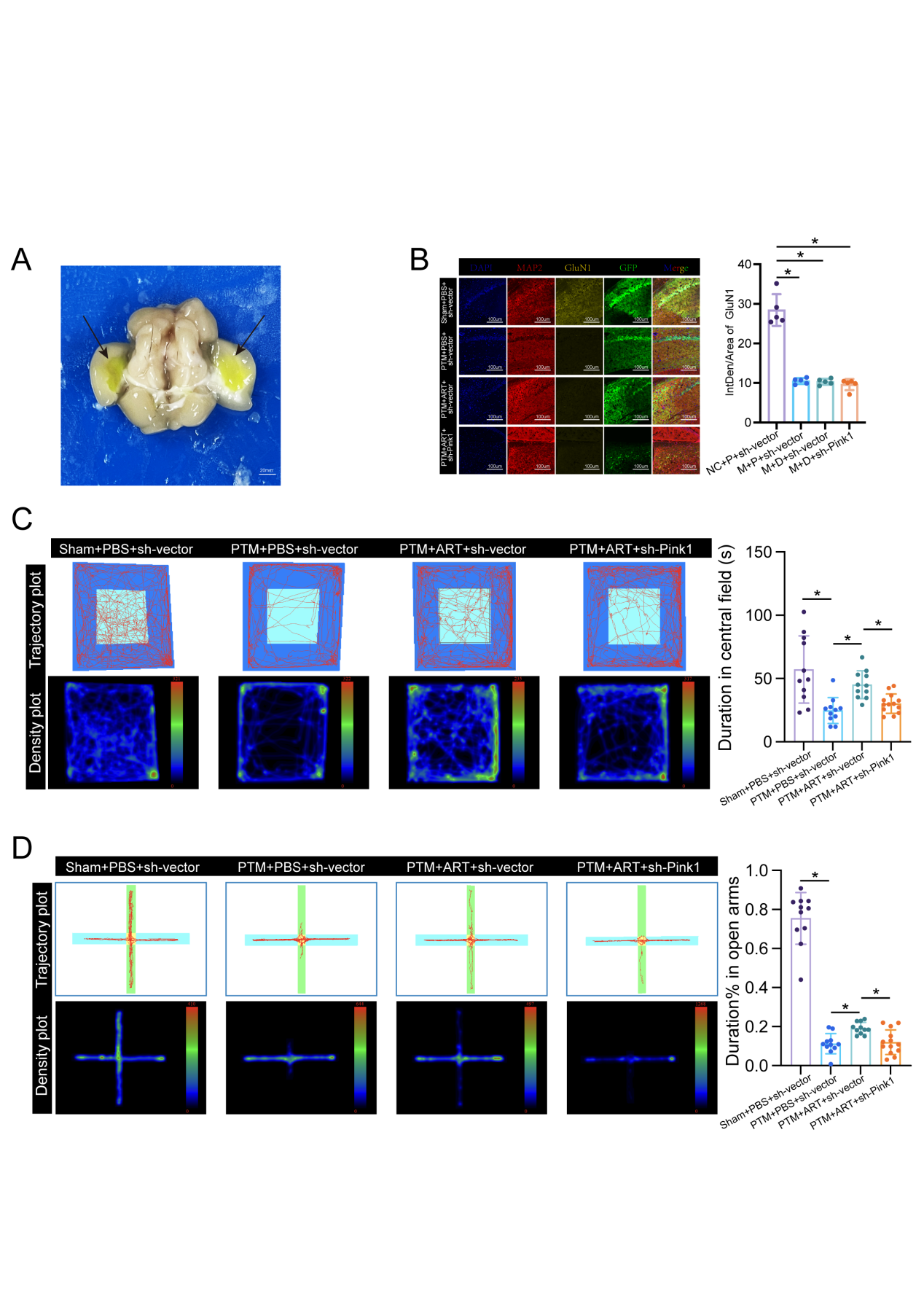
Supplementary Figure**

**Related to Figure 5**

**A**: Macroscopic image of brain tissue from passive transport model mice. The arrows indicate the hippocampal tissue labeled with green fluorescence. **B**: Immunofluorescence staining of hippocampal tissue from the different groups and quantitative statistical analysis of GluN1 fluorescence signals. n=5. DAPI represents the cell nuclei (blue), MAP2 represents microtubule-associated protein 2 (red), GluN1 represents GluN1 receptors in the hippocampal tissue labeled with anti-GluN1 antibody (yellow), and GFP represents hippocampal neurons infected with GFP-labeled virus (green). **C**: Trajectory and density plots illustrating the open field experiments in different groups, along with corresponding quantitative statistical graphs. n=11, 11, 11, and 13 for the sham+PBS, sham+ART, PTM+PBS and PTM+ART groups, respectively. More tracks or a higher density in the central area indicate stronger autonomous movement ability in mice and more active exploratory behavior toward new and different environments. **D**: Trajectory and density plots presenting the results of different groups of mice in the elevated plus maze test, along with corresponding quantitative statistical graphs. n=11, 11, 11, and 13 for the sham+PBS, sham+ART, PTM+PBS and PTM+ART groups, respectively. The horizontal arms represent closed arms, while the vertical arms represent open arms. Sham represents the sham surgery group, PTM represents the passive transport model, and ART represents artemisinin. The sh-vector represents the empty vector, while the sh-PINK1 represents the AAV9 vector capable of downregulating PINK1 expression. *P < 0.05 versus the indicated group. ART represents artesunate
