# Supplementary figures and images for: Exploring the neuroprotective role of artesunate in mouse models of anti-NMDAR encephalitis: insights from molecular mechanisms and transmission electron microscopy
Source: Cell Commun Signal. 2024 May 14;22:269. doi: 10.1186/s12964-024-01652-4 (PMC11094908; doi:10.1186/s12964-024-01652-4)

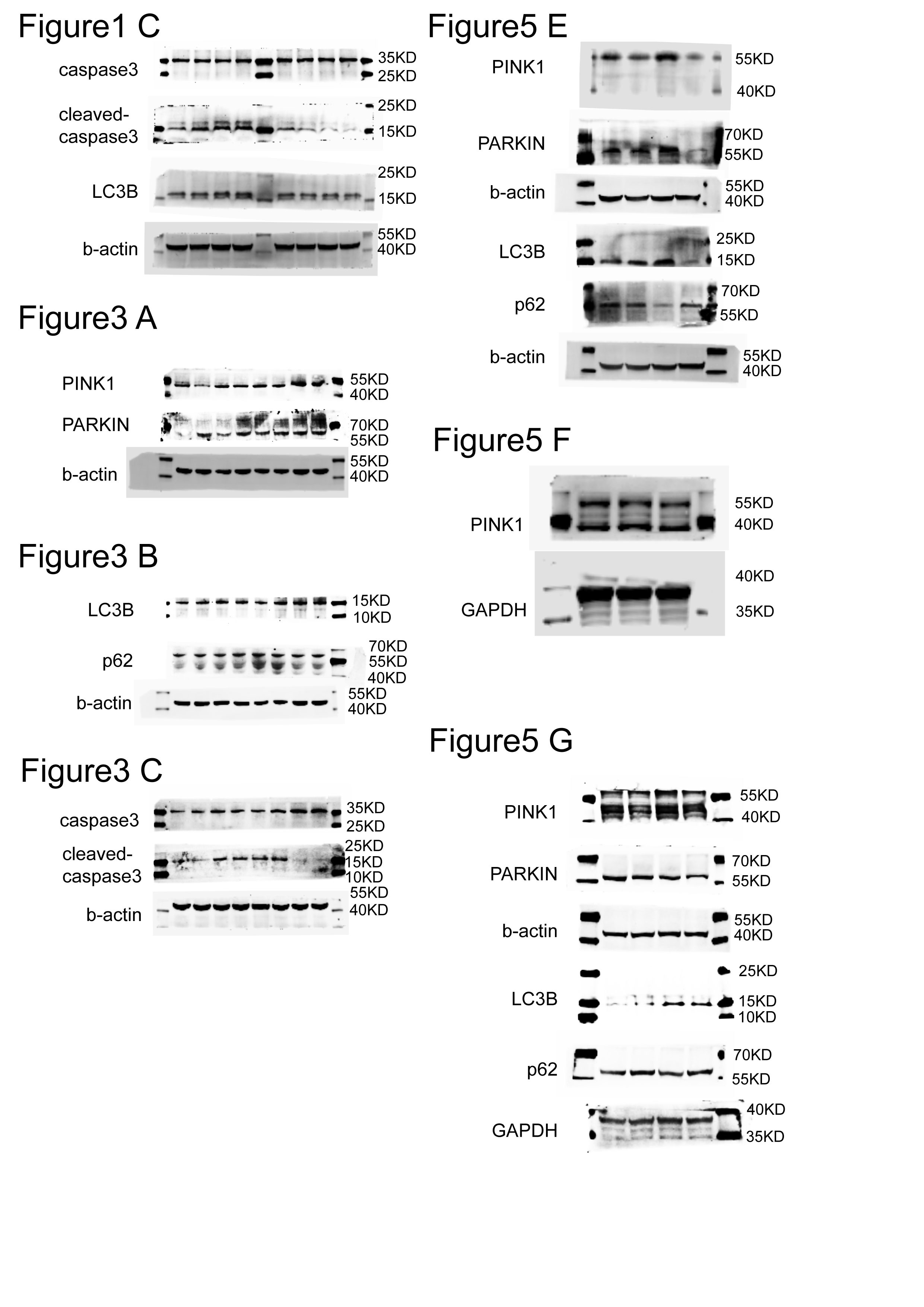

Supplement: Supplementary file 2 — Supplementary Material 2. [file 12964_2024_1652_MOESM2_ESM.tif]
